# Nested interactions between chemosynthetic lucinid bivalves and seagrass promote ecosystem functioning in contaminated sediments

**DOI:** 10.3389/fpls.2022.918675

**Published:** 2022-07-22

**Authors:** Ulisse Cardini, Lazaro Marín-Guirao, Luis M. Montilla, Ugo Marzocchi, Salvatore Chiavarini, Juri Rimauro, Grazia Marina Quero, Jillian M. Petersen, Gabriele Procaccini

**Affiliations:** ^1^Department of Integrative Marine Ecology, Stazione Zoologica Anton Dohrn - National Institute of Marine Biology, Ecology and Biotechnology, Naples, Italy; ^2^Centro Oceanográfico de Murcia, Instituto Español de Oceanografia (IEO-CSIC), Murcia, Spain; ^3^Department of Biology, Center for Water Technology (WATEC), Aarhus University, Aarhus, Denmark; ^4^Division Protection and Enhancement of the Natural Capital - Italian National Agency for New Technologies, Energy and Sustainable Economic Development (ENEA), Rome, Italy; ^5^Institute for Biological Resources and Marine Biotechnology, National Research Council (IRBIM-CNR), Ancona, Italy; ^6^Division of Microbial Ecology, Centre for Microbiology and Environmental Systems Science, University of Vienna, Vienna, Austria

**Keywords:** ecological facilitation, ecosystem restoration, nature-based solutions, chemosynthetic symbioses, *Loripes orbiculatus*, *Cymodocea nodosa*, Bagnoli-Coroglio, sediment contamination

## Abstract

In seagrass sediments, lucinid bivalves and their chemoautotrophic bacterial symbionts consume H_2_S, relying indirectly on the plant productivity for the presence of the reduced chemical. Additionally, the role of lucinid bivalves in N provisioning to the plant (through N_2_ fixation by the symbionts) was hypothesized. Thus, lucinids may contribute to sediment detoxification and plant fitness. Seagrasses are subject to ever-increasing human pressure in coastal environments. Here, disentangling nested interactions between chemosynthetic lucinid bivalves and seagrass exposed to pollution may help to understand seagrass ecosystem dynamics and to develop successful seagrass restoration programs that consider the roles of animal-microbe symbioses. We evaluated the capacity of lucinid bivalves (*Loripes orbiculatus*) to promote nutrient cycling and seagrass (*Cymodocea nodosa*) growth during a 6-week mesocosm experiment. A fully crossed design was used to test for the effect of sediment contamination (metals, nutrients, and hydrocarbons) on plant and bivalve (alone or interacting) fitness, assessed by mortality, growth, and photosynthetic efficiency, and for the effect of their nested interaction on sediment biogeochemistry. Plants performed better in the contaminated sediment, where a larger pool of dissolved nitrogen combined with the presence of other trace elements allowed for an improved photosynthetic efficiency. In fact, pore water nitrogen accumulated during the experiment in the controls, while it was consumed in the contaminated sediment. This trend was accentuated when lucinids were present. Concurrently, the interaction between clams and plants benefitted both organisms and promoted plant growth irrespective of the sediment type. In particular, the interaction with lucinid clams resulted in higher aboveground biomass of *C. nodosa* in terms of leaf growth, leaf surface, and leaf biomass. Our results consolidate the notion that nested interactions involving animal-microbe associations promote ecosystem functioning, and potentially help designing unconventional seagrass restoration strategies that exploit chemosynthetic symbioses.

## Introduction

Seagrasses are habitat-forming marine plants that build the foundation of biodiversity hotspots in coastal marine environments (Heck et al., 2008). However, seagrass ecosystems are under threat due to a variety of human activities, such as coastal exploitation, eutrophication, and climate change (Orth et al., 2006). In many locations, seagrass meadows are becoming fragmented or have already completely disappeared, and have been replaced by bare sediments or by opportunistic macrophytes (Montefalcone et al., 2015). Thus, the restoration of seagrass habitats is often an environmental and economic imperative given that seagrasses provide key ecosystem functions and services (Reynolds et al., 2016), and has recently been recognized as a key action to address the causes of climate change and to mitigate associated effects (Gattuso et al., 2018). However, over the years, many seagrass restoration and/or transplantation programs have been costly and unsuccessful (Cunha et al., 2012; van Katwijk et al., 2016). Possibly, this is because these programs did not take into account factors such as the genetic features of donor populations (Pazzaglia et al., 2021) or the important role of positive species interactions in effectively contributing to ecosystem functioning (Cardinale et al., 2002; Bulleri et al., 2018; Valdez et al., 2020; Gagnon et al., 2021; Malkin and Cardini, 2021; Zhang et al., 2021).

Increasing evidence supports the notion that nested interactions involving animal-microbe associations (also called holobionts) fundamentally contribute to the functioning of diverse marine ecosystems (Pita et al., 2018). The most iconic example is that of coral reefs, where a symbiosis between an animal and a microalgal symbiont forms the basis of some of the most diverse ecosystems on Earth (Muscatine and Porter, 1977). Seagrasses are themselves holobionts associating with a diverse community of microbes which grow on their leaves as epiphytes or inhabit their rhizosphere (Tarquinio et al., 2019). These microbes play fundamental roles in the overall ecosystem functioning. For example, leaf epiphytes were shown to contribute significantly to the plant N needs, by fixing atmospheric N_2_ or converting dissolved organic nitrogen (DON) compounds into bioavailable inorganic forms (DIN) (Agawin et al., 2016; Cardini et al., 2018; Tarquinio et al., 2018). Similarly, sulfate-reducing bacteria (SRB) and other microorganisms in the seagrass rhizosphere significantly contribute to the mineralization of organic N and phosphorus (P), and to anaerobic N_2_ fixation (Welsh, 2000). Recently, Scholz et al. (2021) demonstrated the widespread relationship of cable bacteria growing in association with the root rhizosphere of aquatic plants and seagrasses. Critically, these bacteria can efficiently remove sulfide from sediments and are likely beneficial for the plant (Malkin and Cardini, 2021; Scholz et al., 2021). Other significant positive effects of microorganisms on seagrasses are for example the production of phytohormones, or defense against pathogens or toxic compounds (see Tarquinio et al. (2019) for a review).

Symbioses between macro- or meiofauna and microbes are ubiquitous and highly diverse in seagrass sediments. Sediment microorganisms can benefit a great deal by associating with invertebrates inhabiting this environment. The invertebrate host can provide the microbial symbionts with access to resources that may be unavailable, such as nutrients, or electron donors and acceptors that may not be available simultaneously in the sediment environment (Beinart, 2019). One prominent example of symbioses inhabiting seagrass sediments is the lucinid clams, where a bivalve host associates with sulfur-oxidizing bacteria that are hosted in the animal gills (Taylor and Glover, 2006). This holobiont was suggested to form a positive nested interaction with seagrasses (van der Heide et al., 2012). In this example of a nested ecosystem, the clam and its microbial symbionts are suggested to contribute to the removal of sulfide (toxic to the plant) from the sediments and thus to enhance seagrass growth (Chin et al., 2021). Additionally, a role of the lucinid clam *Loripes orbiculatus* in N provisioning to the seagrass ecosystem was recently proposed, given the ability of the symbionts to also fix atmospheric N_2_ (Petersen et al., 2017; Cardini et al., 2019).

Seagrasses create the conditions for biodiversity hotspots through their role as habitat-forming species; at the same time, efforts that incorporate biodiversity as a means for the restoration of this important ecosystem may be more successful (Williams et al., 2017). Therefore, in this study, we aimed to test the importance of nested interactions between the plant (*Cymodocea nodosa*), the lucinid clams (*Loripes orbiculatus*), and their symbionts, in enhancing seagrass performance and growth in natural vs. contaminated sediments. By means of a mesocosm experiment, we explored the possibility of exploiting nested interactions for successful seagrass restoration strategies. We used a fully crossed design to examine the effect of sediment contamination (metals, nutrients, and hydrocarbons) on plant and bivalve (alone or interacting) fitness, assessed by mortality, growth, and photosynthetic efficiency, and for the effect of their nested interaction on sediment biogeochemistry. We hypothesized that the interaction between *Cymodocea nodosa* and *Loripes orbiculatus* may benefit both organisms in colonizing contaminated sediments and may provide a potential restoration strategy that exploit nested interactions as a nature-based solution in coastal polluted areas.

## Materials and methods

### Collection of sediments, plants, and lucinids

Collection of sediments, plants, and lucinids was carried out at the end of May 2018. Control sediment was collected north of the Gulf of Napoli, at Cape Miseno (40°47′5.75″N–14°4′36.79″E), while polluted sediments were collected within the bay of Bagnoli-Coroglio (40°48′22.10″N–14°9′44.59″E), a coastal area impacted by industrial contamination of hydrocarbons and heavy metals (Morroni et al., 2020). At each site, 150 L of surface sediment (max depth 10 cm) was collected between 5 and 10 m depth by divers using a hand-drag, and immediately transported to the laboratories of the Stazione Zoologica Anton Dohrn (SZN) in Napoli, Italy. *Cymodocea nodosa* plants were collected at Cape Miseno at ~8 m depth. Large fragments of the species were gently uprooted by divers and transported in coolers to the SZN facilities (within 2 h) to be subsequently introduced into the aquaria for plant acclimation. Specimens of *Loripes orbiculatus* were collected by scuba diving in the bay of Fetovaia, Livorno (Italy) from sediments adjacent to a *Posidonia oceanica* meadow (42°43′48″N 10°9′23″E) at ~7 m depth. The bivalves were moved to the HYDRA Institute for Marine Sciences in Fetovaia, prepared for transport in water-tight containers with a good quantity of their surrounding sediment, seawater, and a headspace for gas exchange, and transported to the SZN within 24 h from sampling.

### Experimental setup

*Cymodocea nodosa* fragments of similar size, composed of 1 apical shoot and 8–10 connected vertical shoots, were selected for the experiment. Fragments were fixed to a plastic square mesh (mesh size: 4 cm) with cable ties to be transplanted into 6 l plastic pots (20 × 30 × 15 cm). Three to four *C. nodosa* fragments were fixed to each plastic square mesh to reproduce the plant density of the meadow at the collection site (513 ± 14 shoots m^−2^). The plastic square mesh was fixed to the top of the pots, and thereafter, sediment was carefully poured to allow roots to maintain their vertical position within the sediment. Half of the pots were filled with control sediment and the other half with polluted sediment. Thereafter, 50 lucinid bivalves of similar size (13.2 ± 1.3 mm shell length), equivalent to a realistic density of ~830 individuals m^−2^ (see van der Geest et al., 2020), were transferred onto the sediment of half of the pots to obtain a crossed design. Lucinid clams were left undisturbed and burrowed in the sediment within 8 h. The pots, filled with either polluted or control “Sediment” (factor 1; 2 levels), were thus reconstructed to recreate four types of “Community” (factor 2; 4 levels): only sediment (S), sediment + plant (P), sediment + lucinids (L), and sediment + plant + lucinids (PL). See Supplementary Figure S1 for a graphical representation of the experimental design. Four pots, one for each level of the factor Community, were allocated inside each of the six 500-L experimental tanks (*n* = 3 for each sediment type). See Ruocco et al. (2019) for a description of the aquarium system. The resulting experimental setup was let to acclimate for 1 week under the environmental conditions present *in situ* during sampling (temperature: 24.5°C; salinity: 37.5 psu; maximum noon irradiance: 275 ± 15 μmol m^−2^ s^−1^; 12:12 h light:dark photoperiod). The same conditions were kept during the entire duration of the experiment, which lasted 6 weeks (42 days) in total.

### Chemical characterization of sediments

Sediments chemical characterization was performed as already reported (Armiento et al., 2020; Morroni et al., 2020). Briefly, total organic carbon (TOC) was determined by a Leco CNS 2000 elemental analysis apparatus. Granulometric size distribution determinations were performed on a Micromeritics SediGraph 5100 X-ray particle size analyzer. Major and trace elements were determined by a PerkinElmer Optima 2000DV ICP-OES and an Agilent 7800 ICP-MS, after mineralization by a microwave-assisted acid digestion (Ethos Easy, Milestone). Hg was determined by Automatic solid/liquid Mercury Analyzer (FKV AMA-254). PAHs were analyzed according to EPA 8270D method with an Agilent 7890A-5975C GC–MS system, after extraction according to EPA 3545a method by an Accelerated Solvent Extractor (Dionex ASE 200) and silica gel cleanup (EPA 3630). Hydrocarbons in the C12–C40 range were determined by GC-FID on an Agilent 7820a system after Dionex ASE 200 extraction and Florisil cleanup. Sediment redox potential was characterized in each experimental pot at 5 and 10 cm below the sediment–water interface at the end of the experiment. Five measurements were taken in each pot at each of the selected sediment depths, by inserting a Crison Pt electrode, connected to a portable pH meter (Crison model 507), into the sediments. The electrode was calibrated with a redox standard solution (Crison 468 mV at 25°C) and redox measurements were referred to the standard hydrogen electrode (207 mV) as described in APHA (1992).

### Pore water nutrients

Pore water was collected using metered stainless steel lances (Cardini et al., 2019) at the start and at the end of the experiment. Seawater was retrieved from above the sediment (seawater control), and at 2 and 10 cm below the sediment–water interface. Two 30 ml seawater samples were retrieved from each depth and experimental pot. One sample was filtered onto 0.22 μm PES membrane filters (Merck Millipore), preserved frozen at −20°C, and analyzed for nitrogen oxides (NO_x_) as the sum of nitrate ( ${\mathrm{NO}}_{3}^{-}$ ) and nitrite ( ${\mathrm{NO}}_{2}^{-}$ ), ammonium ( ${\mathrm{NH}}_{4}^{+}$ ), and orthophosphate ( ${\mathrm{PO}}_{4}^{3-}$ ) concentrations on a Continuous Flow Autoanalyzer (Flowsys, Systea) at the SZN laboratories. The other sample was filtered using an acid-washed 50 ml polycarbonate syringe through a pre-combusted 0.7 μm GF/F filter directly into acid-washed 30 ml HDPE sample bottles (Cardini et al., 2015). The sample was then immediately acidified with 80 μl of 18.5% HCl and stored in the dark at 4°C until analysis at the SZN by the high-temperature catalytic oxidation method on a TOC-L Analyzer with a total nitrogen (TN) unit (Shimadzu) for DOC and DON (as the difference of TN and dissolved inorganic nitrogen) quantification. No differences between “Community” levels were detected at the start of the experiment, and t0 data were thus pooled in one group and compared against the “Community” levels at the end of the experiment. Further, no differences were detected between sediment depths, and samples were thus pooled within the respective “Sediment” and “Community” level.

### Plant photophysiology

A diving-PAM fluorometer (Walz, Germany) was used to characterize the functioning of the photosynthetic apparatus at the level of photosystem II (PSII). Chlorophyll fluorescence measurements were taken in two randomly selected *C. nodosa* shoots per experimental pot following Marín-Guirao et al. (2013b). Briefly, basal (F_0_) and maximum fluorescence (F_m_) were measured on whole-night adapted plants by the saturation pulse method to calculate the maximum quantum yield of PSII [(F_v_/F_m_ = (F_m_ − F_0_)/F_m_]. Subsequently, rapid light curves (RLC) were generated on the same shoots after 4 h of illumination in experimental tanks to estimate maximum relative electron transport rates (rel-ETR_max_). Each RLC was composed of 20 s exposure to 9 incremental irradiances. The curve fitting method developed by Jassby and Platt (1976) was used for calculating rel-ETR_max_. Non-photochemical quenching was calculated as NPQ = (F_m_ − F_m_′)/F_m_′; where F_m_′ is the maximum fluorescence of light-adapted leaves obtained from the RLCs. Measurements taken within each pot were averaged to be used as independent replicates (*n* = 3).

### Plant morphology, growth, and mortality

Seagrass growth was measured as leaf elongation and rhizome growth. Leaf elongation was determined by marking the leaves of five randomly selected shoots with a needle 3 weeks after the beginning of the experiment (Zieman, 1974). Marked shoots were collected at the end of the experiment to measure the surface area of newly-formed leaf tissues (cm^2^ shoot^−1^ day^−1^). Total leaf biomass, the number of leaves, and the percentage of the necrotic leaf surface were also determined on marked shoots. Rhizome growth was determined by marking the apical shoot of each plant fragment with plastic ties at the beginning of the experiment. Plant fragments were harvested at the end of the experiment and the newly produced tissues divided into leaves, rhizomes, and roots before being dried and weighed to estimate their biomass. Measurements taken within each pot were averaged to be used as independent replicates (*n* = 3). Finally, all shoots in each experimental pot were counted at the beginning and at the end of the experiment, and the differences normalized to the initial shoot number and expressed as a percentage of net shoot change.

### Lucinid clam mortality and tissue analyses

All *L. orbiculatus* clams were counted at the end of the experiment for determining their mortality rate in the different experimental pots. Additionally, 10 clams at T0 and four clams from each pot at the end of the experiment were selected randomly, measured for their shell length, and dissected for tissue analyses. Symbiont-bearing (gill) tissue and non-symbiotic (host) tissue (i.e., the remaining tissue after removal of the gills) were separated and stored at −20°C to determine the natural ^13^C/^12^C and ^15^N/^14^N ratios of the gill and host tissues as in Cardini et al. (2019). Frozen tissues were freeze-dried for 48 h, ground to fine powder, and weighed into tin capsules. Samples were analyzed for C% and N% and for δ^13^C and δ^15^N by continuous-flow isotope ratio mass spectrometry (IRMS, Isoprime, GV Instruments Ltd) coupled with an elemental analyzer (Costech Instruments).

### Data analysis

Differences in sediment inorganic and organic nutrient concentrations, plant (photochemistry, morphology, and growth), and lucinid clam (mortality) were tested using PERMANOVA tests (Anderson, 2008) with “Sediment” and “Community” as fixed factors. The test for Redox potential additionally included the factor “Depth.” The analysis was conducted using the Euclidean distance as a coefficient of dissimilarity on previously normalized data. Type 3 (partial) sum of squares was used with the unrestricted permutation of raw data (9,999 permutations). These analyses were run using the PERMANOVA tool included in the PRIMER 6+ package. A principal component analysis (PCA) was also performed to explore overall plant responses (photochemistry, morphology and growth) to “Sediment” and “Community” experimental treatments. The isotopic niche spaces of symbionts and hosts were compared among experimental pots analyzing the Bayesian standard ellipse areas (SEA_B_) with the SIBER R package (Jackson et al., 2011; R Core Team, 2021).

## Results

### Sediment geochemistry and pore water nutrients

Both sediments had a similar grain size distribution, characteristic of sandy sediments (Table 1). However, the organic content of the two sediment types differed significantly in both their TOC and DON content, as well as for their DOC:DON ratios (Table 1).

**Table 1:** Sediment organic content and grain size distribution (*n* = 6).

Parameter	Control	Polluted	*p* value
TOC (%)	0.02 ± 0.01	0.36 ± 0.06	<0.01
DOC (μM)	174.57 ± 22.02	166.39 ± 15.99	
DON (μM)	9.65 ± 2.18	35.28 ± 6.53	<0.01
DOC:DON	18.51 ± 2.53	4.82 ± 0.79	<0.01
Gravel: >2 mm (%)	0.30	0.30	
Sand: 2 > 0.063 mm (%)	99.60	99.40	
Silt: <0.063 mm (%)	0.10	0.30	

Abundant elements showed similar concentrations in sediments from both sites (Supplementary Table S1). However, P and Fe were significantly more concentrated in polluted sediments (Table 2). Further, heavy metals and metalloids were significantly more concentrated in polluted sediments (Table 2), with some vastly exceeding environmental quality standards, such as arsenic (As), cadmium (Cd), and lead (Pb). Similarly, hydrocarbon concentrations were high in polluted sediment, with the EPA’s 16 priority pollutant polycyclic aromatic hydrocarbons exceeding by 5-fold the environmental quality standard (Table 2).

**Table 2:** Concentration of key elements and hydrocarbons in the sediment, and environmental quality standard expressed as an annual average values (EQS-AA) according to the IT law 260/2010.

**Element (ppm)**	**Control**	**Polluted**	**EQS-AA**
Fe	20,458 ± 155	102,466 ± 2,794	
P	504 ± 10	4,336 ± 68	
As	21.3 ± 0.6	**75.2 ± 2.9**	12 ± 20%
Cd	0.148 ± 0.011	**0.76 ± 0.04**	0.3 ± 20%
Cr	13.8 ± 2.4	30.9 ± 1.1	50 ± 20%
Cu	6.34 ± 0.40	157 ± 9	
Hg	0.009 ± 0.001	0.220 ± 0.019	0.3 ± 20%
Ni	7.84 ± 1.30	13.6 ± 1.4	30 ± 20%
Pb	31 ± 1	**281 ± 15**	30 ± 20%
**Hydrocarbons (ppb)**	**Control**	**Polluted**	
Σ PAHs (16 priority pollutants EPA)	40.5	**4,450.4**	800 ± 20%
Heavy hydrocarbons (C > 12)	18.5 ± 3.7	155.5 ± 28	

Numbers in bold indicate values exceeding the EQS-AA.

Redox potential (Supplementary Figure S2; Supplementary Table S3) significantly differed between the two analyzed sediment depths (*p* < 0.001) and among communities (*p* < 0.01) but was similar between control and polluted sediments. Sediment redox potential at 5 cm depth was significantly higher in all treatments, almost double that at 10 cm depth. Regardless of depth, the redox potential in the PL community was significantly higher than that in the S community, both in the control (*p* < 0.05) and in the polluted sediment (*p* < 0.01). In the control sediment, lower redox values were found associated with the S community, with values significantly different from the L community (*p* < 0.05). In the polluted sediment, lower redox potentials were associated with the L community, which significantly differed from the redox conditions of the PL community (*p* < 0.05). Across all treatments, the highest mean redox potential was found in PL communities of polluted sediments (102.7 ± 13.7 mV), while the lowest was found in L communities of polluted sediments (26.5 ± 27.3 mV).

Inorganic and organic nutrient concentrations were stable in the aquaria seawater during the experiment (Supplementary Table S2), while they generally increased in the control sediment pore water, regardless of the “Community” level (Supplementary Figure S3; Supplementary Table S3). The same trend was observed in the polluted sediment pore water for ${\mathrm{PO}}_{4}^{3-}$ and DOC, although DOC increased significantly more in the P and PL community compared to the other treatments (Supplementary Figure S3). Conversely, we detected a significant decrease in ${\mathrm{NH}}_{4}^{+}$ and DON concentrations in the polluted sediment, particularly in the PL community (Supplementary Figure S3).

### Plant photophysiology

**Figure 1:**
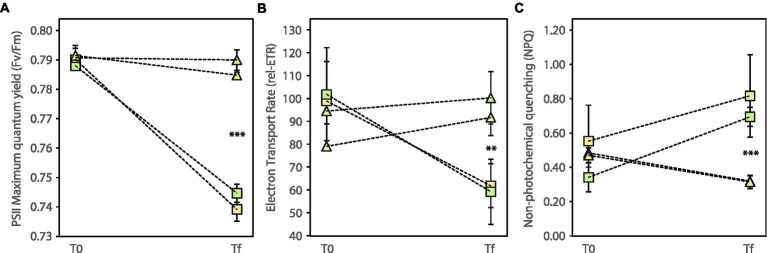
Evolution of chlorophyll a fluorescence parameters. **(A)** Maximum quantum yield of PSII (F_v_/F_m_), **(B)** electron transport rate (rel-ETR), **(C)** non-photochemical quenching (NPQ) at the beginning (T0) and end (Tf) of the experiment (±SE, *n* = 3). Colors indicate absence (yellow) or presence (green) of the interaction with lucinid clams. Symbols are used to indicate control (quadrats) or polluted (triangles) sediment. Asterisks indicate significant differences in the factor “Sediment” at Tf (^**^p < 0.01; ^***^p < 0,001); see Supplementary Table S4 for the statistics.

At the end of the experiment, the photochemical efficiency of plants growing in polluted sediments was significantly higher than the efficiency of plants in control sediments (Figure 1; Supplementary Table S4). These plants also showed significantly higher values of electron transport rate (rel-ETR) but lower values of non-photochemical quenching (NPQ) (Figure 1; Supplementary Table S4). There was no indication of an effect of the community type on the photophysiology of *C. nodosa*.

### Plant morphology, growth, and mortality

**Figure 2:**
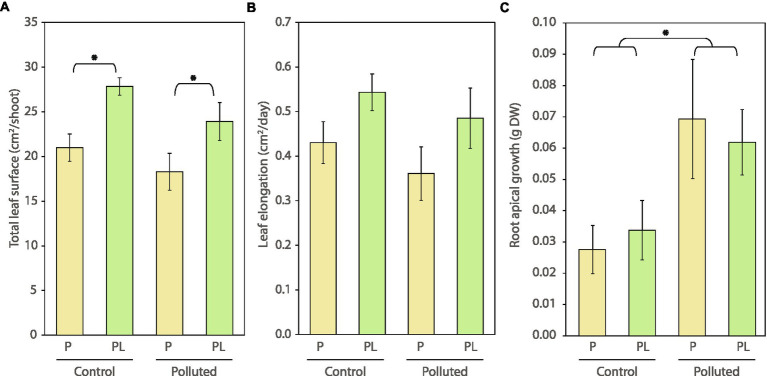
Plant morphology and growth. **(A)** Total leaf surface, **(B)** leaf elongation, **(C)** root apical growth of *C. nodosa* at the end of the experiment (±SE, *n* = 3). Levels of the factor “Community” are identified with letters as indicated in the methods. Colors indicate absence (yellow) or presence (green) of the interaction with lucinid clams. Asterisks (^*^p < 0.05) indicate significant differences; see Supplementary Tables S5, S6 for the statistics.

The total leaf surface area of *C. nodosa* interacting with *L. orbiculatus* was significantly higher irrespective of sediment pollution (Figure 2; Supplementary Table S5). These plants also showed a trend of higher leaf elongation and leaf biomass compared to plants growing in the absence of lucinid clams, but differences were deemed not significant by the statistical test (Figure 2; Supplementary Figure S4; Supplementary Table S5). New apical growth was measured for the root, rhizome, and leaf portion of the plant. Neither rhizome nor leaf apical growth showed significant differences among the experimental pots (Supplementary Figure S5; Supplementary Table S6). However, there was a significant effect of the sediment type, regardless of the interaction with lucinids, on the growth of the apical roots (Figure 2; Supplementary Table S6). At the same time, there was a significant increase of necrotic tissue in *C. nodosa* from the polluted compared to the control sediment, regardless of the presence of the clams, while all plots showed a positive net shoot change during the course of the experiment regardless of the experimental treatment (Supplementary Figure S4; Supplementary Table S5).

**Figure 3:**
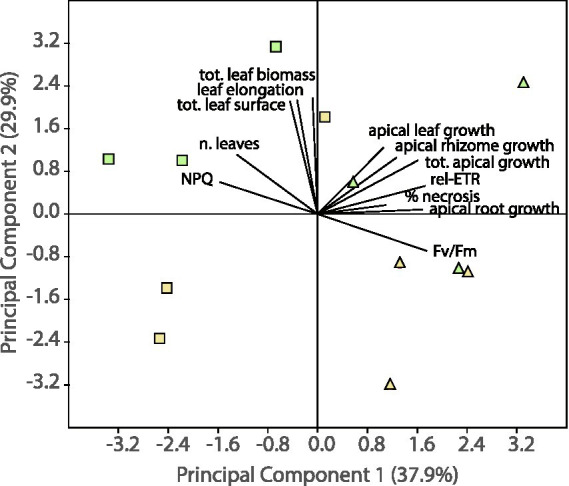
Principal component analysis testing for multivariate changes in plant variables among treatment levels. Colors indicate absence (yellow) or presence (green) of the interaction with lucinid clams. Symbols are used to indicate control (quadrats) or polluted (triangles) sediment. Note that the per cent variation explained by the principal components is indicated in the graph and refers to the fraction of the total variance explained by each axis or principal component (i.e., PC1 and PC2).

In a principal component analysis (Figure 3), sediment type (control vs. polluted) segregated samples along axis 1 (37.9% of total variance), which was mainly correlated with photochemical parameters and the newly produced tissues by apical growth. Conversely, the community type (P vs. PL) segregated samples along axis 2 (29.9% of total variance). This axis was mainly correlated with leaf growth and shoot size (in terms of both leaf surface and leaf biomass).

### Lucinid clams

**Figure 4:**
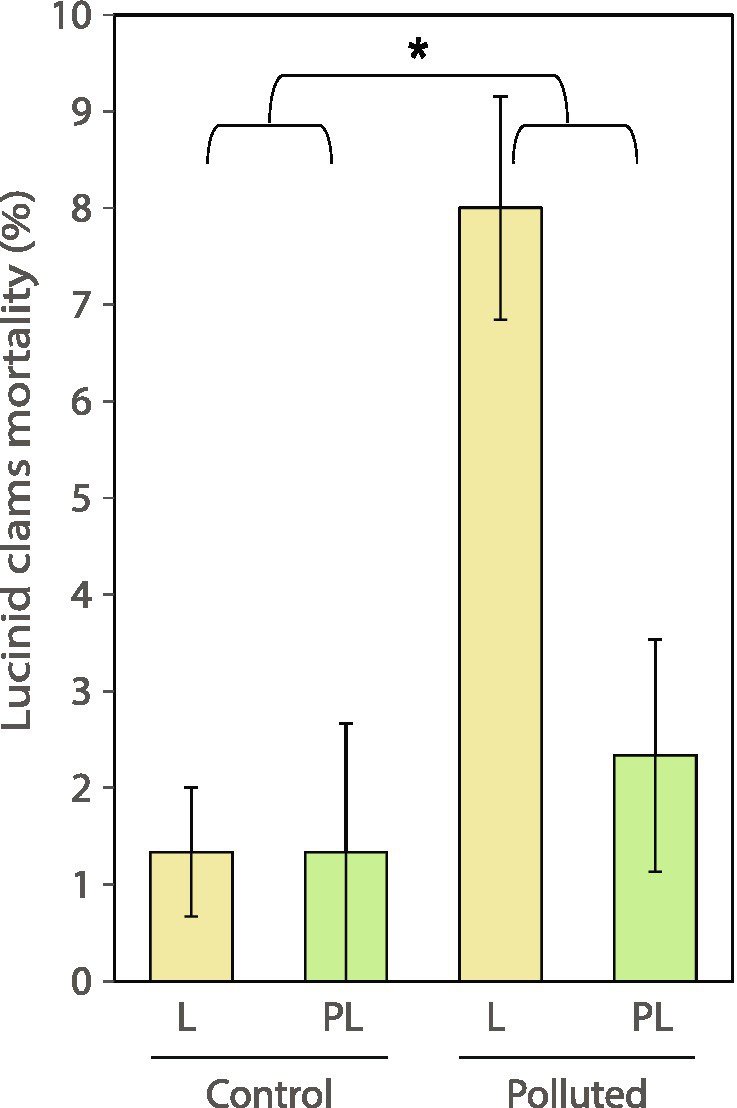
Lucinid clams mortality (±SE, *n* = 3). Levels of the factor “Community” are identified with letters as indicated in the methods. Colors indicate absence (yellow) or presence (green) of the interaction with the plant. Asterisks (^*^p < 0.05) indicate significant differences; see Supplementary Table S6 for the statistics.

**Figure 5:**
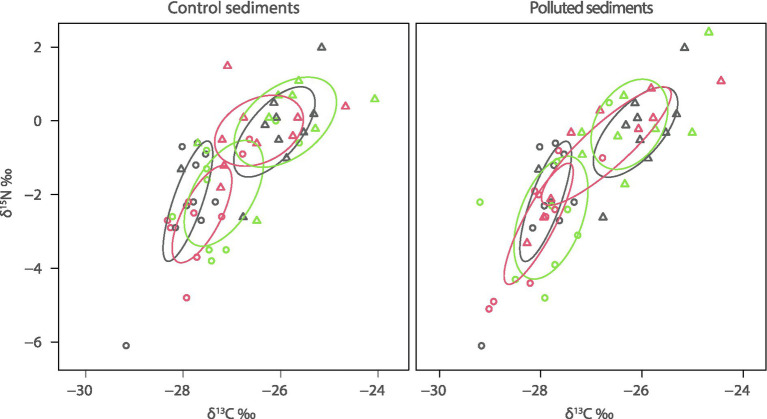
Isotopic niche of lucinid tissues exposed to the different experimental conditions. The lines enclose the Bayesian standard ellipse area (SEA_B_). Initial (T0) samples are represented in grey, samples from the L treatment are in red, while those from the PL treatment are shown in green. Triangles are used for symbiont-free (rest) and circles for symbiont-hosting (gill) lucinid clam tissues. See Supplementary Figure S6 for the density plot of SEA_B_.

*Loripes orbiculatus* showed significantly more mortality in the polluted sediment (Figure 4; Supplementary Table S6) compared to the control sediment (*p* < 0.05), approaching a value of 10% where the plant was not present. The isotopic niche of *L. orbiculatus* sampled at the beginning of the experiment showed a differentiation between symbiont-free (rest) and symbiont-hosting (gill) lucinid clam tissues, with the latter having more negative δ^13^C and δ^15^N values (Figure 5) and in SEA_B_ (Supplementary Figure S6). At the end of the experiment, the same pattern was generally maintained in all treatments. SEA_B_ of *L. orbiculatus* tissues overlapped significantly between the beginning and the end of the experiment, except for symbiont-free (rest) tissues in the L treatment of the polluted sediment (Figure 5). These samples also showed the largest SEA_B_ (Supplementary Figure S6). The interaction of *L. orbiculatus* with the plant also caused larger SEA_B_, regardless of the sediment type (Supplementary Figure S6).

## Discussion

Here, we found evidence that nested interactions between chemosynthetic lucinid bivalves and seagrass promote ecosystem functioning, and that these interactions can play a role in the capacity of the mutualistic consortium to resist stress and grow in polluted sediments.

### *Cymodocea nodosa* is tolerant to pollution

Our study confirms that *C. nodosa* is highly plastic at the morphological level. This species can in fact rapidly change the plant architecture modifying the ratio of above- vs. below-ground biomass depending on environmental conditions and resource availability (Perez et al., 1994; Marín-Guirao et al., 2018). Our study demonstrates that this plant is resistant to high doses of pollution deriving from the massive industrial contamination by trace metals and hydrocarbons found in the Bagnoli area (Armiento et al., 2020; Morroni et al., 2020).

In our experiment, *C. nodosa* plants exposed to pollution showed increased photosynthetic efficiency and apical growth. Nutrients in *C. nodosa* are mainly taken up through the root system, with leaf uptake dominant only when seawater concentrations suddenly increase after a nutrient pulse (Alexandre and Santos, 2020). In particular, ammonium is the preferential nitrogen source for *C nodosa*, while amino acids can also represent a large fraction of this species’ N demand (Alexandre and Santos, 2020). In our study, the higher availability of nutrients (and particularly of ammonium and dissolved organic nitrogen) in polluted sediments likely contributed to increased uptake rates and stimulation of plant roots growth.

Other important macronutrients (P) and as well as micronutrients/trace metals (e.g., Fe, Cu, Mn, and Zn) that are used as co-factors in photosynthesis were significantly more abundant in polluted sediments, possibly explaining the more efficient use of light for *C. nodosa* plants. At the same time, our lower NPQ in plants exposed to pollution suggests that *C. nodosa* activated responses at the physiological level that enhance acclimation and plant tolerance to stress (Marín-Guirao et al., 2013a). Indeed, a recent study showed for NPQ a biphasic dose–response pattern typical of hormesis in *C. nodosa* exposed to ZnO nanoparticles (Malea et al., 2019).

In a different study, *C. nodosa* meadows growing on mining-impacted sediments were more dense and lush than meadows on control sediments (Marín-Guirao et al., 2005), further demonstrating the capacity of *C. nodosa* to tolerate stress from heavy metals. Many of these metals are accumulated by the plant if bioavailable and not sulfide-bound. Unfortunately, neither metal accumulation in plant tissues nor sulfide concentrations were quantified in this study, making it impossible to speculate whether the plant was able to cope with pollution because of their low bioavailability or despite their accumulation. Notwithstanding, it appears clear that *C. nodosa* is resistant to stress deriving from heavy metal and hydrocarbon contamination, at least when this is accompanied by a significant increase in macro and micronutrient availability that boost plant growth.

### *Loripes orbiculatus* response depends on seagrass presence

The lucinid bivalve *L. orbiculatus* was susceptible to pollution, as indicated by the significantly higher mortality rate of clams burrowing in polluted sediment, particularly for those animals maintained in the absence of the plant. Further, in this study, we explored the response of the *L. orbiculatus* chemosynthetic symbiosis to pollution and interaction with *C. nodosa* by quantifying the isotopic niche width of the symbiotic vs. non-symbiotic clam tissues.

The isotopic niche has become an established concept in ecology because stable isotope ratios in consumer tissues are tightly linked to those in their diet (Jackson et al., 2011), offering a potentially powerful way to investigate ecological niches and trophic interactions (Yeakel et al., 2016). Recently, the method was used to also look at trophic interactions in chemosynthetic symbioses (Cardini et al., 2019). Stress-induced variability in physiological status can induce changes in isotopic niche width. For example, greater isotopic niche estimates were derived for the deposit-feeding amphipod *Monoporeia affinis* exposed to sediments contaminated with polychlorinated biphenyls, heavy metals, chlorophenols, and polycyclic aromatic hydrocarbons (Karlson et al., 2018).

Similarly, in our study, greater isotopic niche estimates were derived for non-symbiotic tissues of *L. orbiculatus* exposed to the polluted sediments, a result which is consistent with the increase in stress-induced mortality for these clams and the concomitant decrease in redox potential as a likely result of clam mortality and decay. The symbiotic (gill) tissues of clams exposed to polluted sediments showed comparable isotopic niche widths compared to the control group, suggesting that the microbial partners (hosted in the bivalve gills) may remain little affected in those individuals that overcome the stressful conditions.

### Nested interactions promote ecosystem functioning

The association between lucinid bivalves and seagrass was suggested to function as a tripartite mutualism (van der Heide et al., 2012). In this system, the plant provides organic matter, which is respired by sulfate-reducing bacteria in anoxic sediments leading to formation of hydrogen sulfide, the energy source needed by the clam’s chemosynthetic bacteria. At the same time, radial oxygen loss by the seagrass roots facilitates the clam’s respiration. In return, the clam symbionts oxidize hydrogen sulfide back to sulfate, preventing a potential build-up of the powerful phytotoxin in sediments (Lamers et al., 2013) and thus, sulfide intrusion into the seagrass with potential to induce plant starvation and mortality (Holmer and Hasler-Sheetal, 2014). This mutualism was recently also verified in a field survey in a temperate lagoon system (van der Geest et al., 2020).

**Figure 6:**
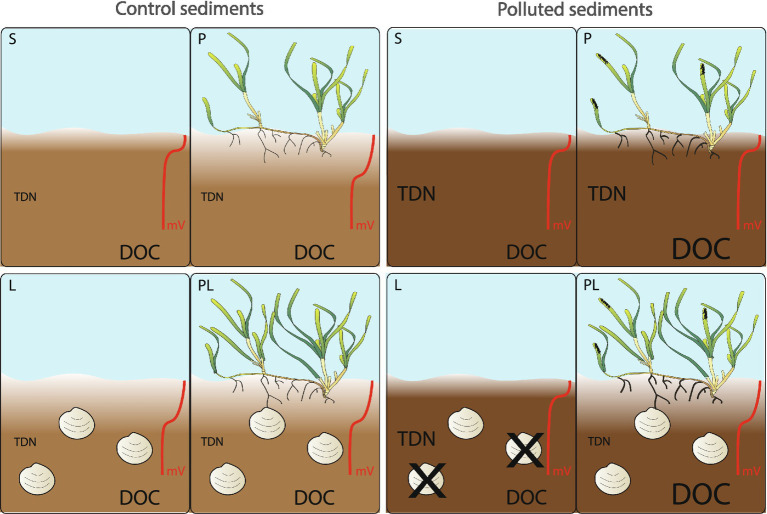
Conceptual model of facilitation of the seagrass *Cymodocea nodosa* by the lucinid clam *Loripes orbiculatus*, in control (left) vs. polluted (right) sediments. (S) represents pots with only sediment; (P) are pots with seagrass; (L) are pots with lucinids; (PL) represents pots with the plant and lucinids. Presence of lucinids resulted in increased above-ground biomass (more leaves) irrespective of the sediment type. Conversely, root apical growth (thicker roots) increased in polluted sediments, regardless of lucinid clams. Lucinid mortality was high in the polluted sediment (black crosses), but only when the plant was absent, while the percentage of plant necrotic tissue (black markings on leaves) were higher in the polluted sediment regardless of lucinids. The interaction of plants and lucinids significantly improved sediment conditions as shown by the increase in redox potential (mV) and decrease (consumption) of total dissolved nitrogen (TDN) in the polluted sediment, where the plant released large quantities of dissolved organic carbon (DOC). Seagrass symbol credit: Integration and Application Network (https://ian.umces.edu/media-library).

Our experiment seems to confirm these studies and the presence of ecological facilitation, irrespective of sediment type. While a longer duration of the experiment and higher replication would have likely resulted in lower variability and clearer differences among the treatments, the experiment clearly showed an effect of both pollution and the interaction between *L. orbiculatus* and *C. nodosa* on some of the investigated variables (Figure 6). The interaction of plants and lucinids significantly improved sediment oxic conditions as shown by the increase of redox potential. At the same time, *C. nodosa* enriched sediment pore water in dissolved organic carbon, a potential food source for sediment sulfate-reducers. The interaction with lucinid clams further resulted in higher aboveground biomass of *C. nodosa* in terms of leaf growth, leaf surface and leaf biomass, similar to the findings of van der Geest et al. (2020) for *L. orbiculatus* and *Zostera noltii* in the Thau lagoon, France.

Our study further demonstrates that the interaction between the plant and lucinid clams facilitates the consortium, especially in heavily contaminated sediments. In these sediments, the interaction with lucinids turned the plant treatment into a sink for nitrogen (both ammonium and dissolved organic nitrogen), suggesting a more efficient uptake of this nutrient when the plant and the clam are associated. While the specific mechanisms involved are difficult to pinpoint, our results seem to support the notion of a role in nitrogen cycling for *L. orbiculatus* as indicated by Cardini et al. (2019).

Further, we found that *L. orbiculatus* isotopic niche is larger (both for the animal host and for the symbiont) when associated with the plant, regardless of the type of sediment. This was previously linked to the flexible nutritional mutualism of *L. orbiculatus,* in which the clam host and its symbionts cycle between a looser trophic association and a tight chemoautotrophic partnership, changing nutritional strategy according to the environmental conditions (Cardini et al., 2019). Importantly, the present study further shows how the remarkable flexibility of this chemosynthetic symbiosis allows it to withstand heavy pollution if associated with a seagrass partner, the plant in turn benefitting and building more aboveground biomass in the presence of the clam.

## Conclusion

Harnessing positive species interactions as a tool for restoration of degraded systems, or to counteract climate-driven loss of coastal biodiversity, is urgently needed (Bulleri et al., 2018). In particular, plant–bivalve interactions have been suggested to facilitate foundation species such as seagrasses, possibly helping to increase the success of restoration efforts (Gagnon et al., 2020). However, studies that mechanistically test specific interactions for their capacity to improve resistance of the whole consortium of organisms to anthropogenic stress are lacking. In this study, we showed that the interaction between *C. nodosa* and *L. orbiculatus* favors both organisms in colonizing highly polluted sediments from the Bagnoli-Coroglio area, promoting growth and resilience of the foundation species. Thus, co-restoration of *C. nodosa* and *L. orbiculatus* may be used in heavily impacted sites where other options are prone to failure and may improve restoration success leading to recovery of associated biodiversity, functioning and ecosystem services.

## Data availability statement

The original contributions presented in the study are included in the article/Supplementary material, further inquiries can be directed to the corresponding authors.

## Author contributions

UC, LM-G, and GP conceived the ideas and designed methodology. UC, LM-G, JP, and GP contributed to the sampling of biological material. UC, LM-G, GP, and GQ performed the experiment and collected the data. UC, LM-G, LMM, UM, SC, and JR analyzed the data. UC and LM-G led the writing of the manuscript. All authors contributed critically to the drafts and gave final approval for publication.

## Funding

This study was supported by the project ABBaCo funded by the Italian Ministry for Education, University and Research (grant number C62F16000170001). Partial support was provided by the project Marine Hazard (PON03PE_00203_1, Italian Ministry of Education), the project Assemble Plus (EU-FP7) to GP, a VRG grant to JP from the Vienna Science and Technology Fund (WWTF), the project INBALANCE (LMT, grant 09.3.3-LMT-K-712-01-0069) funded by the European Social Fund, and the Grundfos Foundation (UM). LMM was supported by a PhD fellowship funded by the Stazione Zoologica Anton Dohrn (Open University – Stazione Zoologica Anton Dohrn PhD Program).

## Conflict of interest

The authors declare that the research was conducted in the absence of any commercial or financial relationships that could be construed as a potential conflict of interest.

## Publisher’s note

All claims expressed in this article are solely those of the authors and do not necessarily represent those of their affiliated organizations, or those of the publisher, the editors and the reviewers. Any product that may be evaluated in this article, or claim that may be made by its manufacturer, is not guaranteed or endorsed by the publisher.
