# Supplementary material for: Nested interactions between chemosynthetic lucinid bivalves and seagrass promote ecosystem functioning in contaminated sediments
Source: Front Plant Sci. 2022 Jul 22;13:918675. doi: 10.3389/fpls.2022.918675 (PMC9355091; doi:10.3389/fpls.2022.918675)
Supplement: Supplementary file 1 [file Data_Sheet_1.docx]

Supplementary Material

# Supplementary Figures and Tables

**Table S1.** Concentration of elements and hydrocarbons in the two sediments (control vs polluted).

| **Element (ppm)** | **Control** | **Polluted** |
| --- | --- | --- |
| Mg | 8,104 ± 336 | 14,660 ± 570 |
| Ca | 59,836 ± 1,000 | 44,442 ±1,315 |
| Na | 21,508 ± 192 | 21,334 ± 396 |
| K | 40,712 ± 371 | 45,293 ±882 |
| Fe | 20,458 ± 155 | 102,466 ± 2,794 |
| P | 504 ± 10 | 4,336 ± 68 |
| Mn | 889 ± 4 | 1,198 ± 19 |
| Al | 64,316 ± 271 | 72,633 ± 1,668 |
| As | 21.3 ± 0.6 | 75.2 ± 2.9 |
| Cd | 0.148 ± 0.011 | 0.76 ± 0.04 |
| Cr | 13.8 ± 2.4 | 30.9 ± 1.1 |
| Cu | 6.34 ± 0.40 | 157 ± 9 |
| Hg | 0.009 ± 0.001 | 0.220 ± 0.019 |
| Ni | 7.84 ± 1.30 | 13.6 ± 1.4 |
| Pb | 31 ± 1 | 281 ± 15 |
| V | 47 ± 1 | 113 ± 5 |
| Zn | 45 ± 2 | 703 ± 26 |
| **Polycyclic Aromatic Hydrocarbons (ppb)** | **Control** | **Polluted** |
| Naphthalene | 0.27 ± 0.06 | 50.3 ± 9.6 |
| Acenaphthylene | < 0.1 | 59.6 ± 7.8 |
| Acenaphthene | < 0.1 | 10.6 ± 1.7 |
| Fluorene | < 0.1 | 17.6 ± 2.1 |
| Pheenanthrene | 2.11 ± 0.23 | 229.8 ± 20.7 |
| Anthracene | 0.16 ± 0.02 | 133.4 ± 14.7 |
| Fluoranthene | 3.35 ± 0.30 | 694.6 ± 48.6 |
| Pyrene | 2.8 ± 0.28 | 546.1 ± 43.7 |
| Benzo[*a*]antracene | 17.6 ± 1.9 | 383.7 ± 34.5 |
| Crysene | 2.22 ± 0.33 | 281.9 ± 36.7 |
| Benzo[*b*]fluoranthene | 1.98 ± 0.20 | 318.0 ± 25.4 |
| Benzo[*k*]fluoranthene | 1.13 ± 0.15 | 157.0 ± 17.3 |
| Benzo[*a*]pyrene | 2.48 ± 0.33 | 551.9 ± 38.6 |
| Indeno[1,2,3-*cd*]pyrene | 2.63 ± 0.26 | 507.9 ± 40.6 |
| Dibenz[*a,h*]anthracene | 1.14 ± 0.16 | 137.4 ± 16.5 |
| Benzo[*ghi*]perylene | 2.65 ± 0.29 | 370.5 ± 33.3 |
| Σ PAH (EPA 16 list) | 40.5 | 4450.4 |
| Benzo[*j*]fluoranthene | 1.00 ± 0.11 | 125.3 ± 11.3 |
| Benzo[*e*]pyrene | 2.75 ± 0.28 | 413.9 ± 33.1 |
| C12-C40 Hydrocarbons | 18.5 ± 3.7 | 155.5 ± 28 |

**Table S2.** Inorganic and organic nutrient concentrations (µM) in the seawater of the experimental aquaria. Results are reported as averages (n = 6) ± standard deviations of T0 and Tfinal, since no significant differences were detected between time intervals.

| Treatment | NH_4_^+^ | NO_X_ | PO_4_^3-^ | DOC | DON |
| --- | --- | --- | --- | --- | --- |
| Control | 1.46 ± 0.31 | 9.43 ± 2.38 | 0.13 ± 0.05 | 203.8 ± 66.5 | 9.43 ± 3.83 |
| Polluted | 1.44 ± 0.19 | 9.74 ± 3.60 | 0.17 ± 0.01 | 238.4 ± 83.0 | 10.09 ± 3.31 |

**Table S3.** Sediment inorganic and organic nutrient concentrations, and sediment redox potential (Eh). Pair-wise tests for the term 'Sediment x Treatment' for levels pairs of the factor 'Treatment'. See the Methods section for details on the analyses. *, P(perm)<0.05; **, P(perm)<0.01; ***, P(perm)<0.001.

|  |  | **NH_4_^+^** | | | | | **NO_X_** | | | | | **PO_4_^3-^** | | | | | **DOC** | | | | | **DON** | | | | | **Eh** | | | |
| --- | --- | --- | --- | --- | --- | --- | --- | --- | --- | --- | --- | --- | --- | --- | --- | --- | --- | --- | --- | --- | --- | --- | --- | --- | --- | --- | --- | --- | --- | --- |
| **Sediment** | **Community** | **T0** | **S** | **L** | **P** | **PL** | **T0** | **S** | **L** | **P** | **PL** | **T0** | **S** | **L** | **P** | **PL** | **T0** | **S** | **L** | **P** | **PL** | **T0** | **S** | **L** | **P** | **PL** | **S** | **L** | **P** | **PL** |
| **Control** | **T0** |  |  |  |  |  |  |  |  |  |  |  |  |  |  |  |  |  |  |  |  |  |  |  |  |  |  |  |  |  |
|  | **S** |  |  |  |  |  | * |  |  |  |  | * |  |  |  |  | ** |  |  |  |  | *** |  |  |  |  |  |  |  |  |
|  | **L** | *** |  |  |  |  | ** |  |  |  |  | * |  |  |  |  | *** |  |  |  |  | *** |  |  |  |  | * |  |  |  |
|  | **P** | *** |  |  |  |  |  |  |  |  |  | ** |  |  |  |  | ** |  |  |  |  | *** |  |  |  |  |  |  |  |  |
|  | **PL** | ** |  |  | * |  | ** |  |  |  |  | *** |  |  |  |  | *** |  |  |  |  | *** |  |  |  |  | * |  |  |  |
| **Polluted** | **T0** |  |  |  |  |  |  |  |  |  |  |  |  |  |  |  |  |  |  |  |  |  |  |  |  |  |  |  |  |  |
|  | **S** | ** |  |  |  |  |  |  |  |  |  | * |  |  |  |  | *** |  |  |  |  | * |  |  |  |  |  |  |  |  |
|  | **L** | ** |  |  |  |  |  |  |  |  |  | * |  |  |  |  | *** |  |  |  |  |  |  |  |  |  |  |  |  |  |
|  | **P** |  |  |  |  |  |  |  |  |  |  | *** |  |  |  |  | *** |  | * |  |  | * |  |  |  |  |  |  |  |  |
|  | **PL** | *** | * |  | * |  |  |  |  |  |  | * |  |  |  |  | *** |  | * |  |  | ** |  | ** |  |  | ** | * |  |  |

**Table S4.** PERMANOVA table of results assessing the effect of “Sediment” and “Community” on photochemical variables derived from chlorophyll a fluorescence measurements.

| **Variable** | **Treatment** | **df** | **MS** | **Pseudo-F** | **P(perm)** | **P(MC)** |
| --- | --- | --- | --- | --- | --- | --- |
| Fv/Fm | Sediment | 1 | 6.67 | 257.73 | **0.0025** | **0.0001** |
|  | Community | 1 | 0.00 | 0.01 | 0.9186 | 0.9257 |
|  | SexCo | 1 | 0.09 | 3.65 | 0.1033 | 0.0937 |
| rel-ETR | Sediment | 1 | 437.01 | 8.55 | **0.0195** | **0.0175** |
|  | Community | 1 | 11.11 | 0.22 | 0.6785 | 0.6757 |
|  | SexCo | 1 | 1.55 | 0.03 | 0.9285 | 0.9294 |
| NPQ | Sediment | 1 | 1220.30 | 24.11 | **0.0026** | **0.0007** |
|  | Community | 1 | 5.17 | 0.10 | 0.8624 | 0.8445 |
|  | SexCo | 1 | 4.08 | 0.08 | 0.9002 | 0.8719 |

**Table S5.** PERMANOVA table of results assessing the effect of “Sediment” and “Community” on morphology and growth of the plant.

| **Variable** | **Treatment** | **df** | **MS** | **Pseudo-F** | **P(perm)** | **P(MC)** |
| --- | --- | --- | --- | --- | --- | --- |
| Tot leaf surface | Sediment | 1 | 42.88 | 3.09 | 0.1359 | 0.1146 |
|  | Community | 1 | 145.26 | 10.47 | **0.0137** | **0.0105** |
|  | SexCo | 1 | 0.55 | 0.04 | 0.8994 | 0.8825 |
| Leaf elongation | Sediment | 1 | 0.008 | 1.42 | 0.2837 | 0.2720 |
|  | Community | 1 | 0.024 | 4.45 | 0.0737 | 0.0712 |
|  | SexCo | 1 | 0.000 | 0.03 | 0.8529 | 0.8624 |
| Leaf biomass | Sediment | 1 | 0.003 | 2.52 | 0.1588 | 0.1548 |
|  | Community | 1 | 0.004 | 4.22 | 0.0768 | 0.0702 |
|  | SexCo | 1 | 0.000 | 0.11 | 0.7434 | 0.7412 |
| N. leaves | Sediment | 1 | 0.009 | 5.01 | 0.0566 | 0.0543 |
|  | Community | 1 | 0.003 | 1.48 | 0.2729 | 0.2611 |
|  | SexCo | 1 | 0.001 | 0.34 | 0.6185 | 0.5808 |
| Necrotic tissue | Sediment | 1 | 5.19 | 7.69 | **0.0264** | **0.0231** |
|  | Community | 1 | 0.92 | 1.37 | 0.2811 | 0.2677 |
|  | SexCo | 1 | 0.95 | 1.41 | 0.2996 | 0.2734 |
| Net shoot change | Sediment | 1 | 0.23 | 0.37 | 0.5599 | 0.5581 |
|  | Community | 1 | 0.30 | 0.47 | 0.5172 | 0.5141 |
|  | SexCo | 1 | 0.27 | 0.42 | 0.5401 | 0.5287 |

**Table S6.** PERMANOVA table of results assessing the effect of “Sediment” and “Community” on the growth of the apical portions of the plant, and on clam mortality.

| **Variable** | **Treatment** | **df** | **MS** | **Pseudo-F** | **P(perm)** | **P(MC)** |
| --- | --- | --- | --- | --- | --- | --- |
| Apical Leaf growth | Sediment | 1 | 1.0E-03 | 0.27 | 0.6068 | 0.6183 |
|  | Community | 1 | 3.5E-03 | 0.90 | 0.3635 | 0.3659 |
|  | SexCo | 1 | 5.5E-04 | 0.14 | 0.7199 | 0.7150 |
| Apical rhizome growth | Sediment | 1 | 2.7E-03 | 3.14 | 0.1057 | 0.1189 |
|  | Community | 1 | 1.7E-03 | 1.94 | 0.2046 | 0.2059 |
|  | SexCo | 1 | 1.2E-03 | 1.39 | 0.2901 | 0.2714 |
| Apical root growth | Sediment | 1 | 2.0E-02 | 9.02 | **0.0207** | **0.0166** |
|  | Community | 1 | 1.8E-05 | 0.01 | 0.9336 | 0.9297 |
|  | SexCo | 1 | 6.1E-04 | 0.28 | 0.5979 | 0.6214 |
| Tot apical growth | Sediment | 1 | 1.4E-02 | 3.01 | 0.1267 | 0.1230 |
|  | Community | 1 | 4.2E-03 | 0.87 | 0.3643 | 0.3745 |
|  | SexCo | 1 | 2.4E-04 | 0.05 | 0.8209 | 0.8340 |
| Clam mortality | Sediment | 1 | 75.00 | 8.33 | **0.0325** | **0.0197** |
|  | Community | 1 | 8.33 | 0.93 | 0.3673 | 0.3569 |
|  | SexCo | 1 | 8.33 | 0.93 | 0.3779 | 0.3610 |


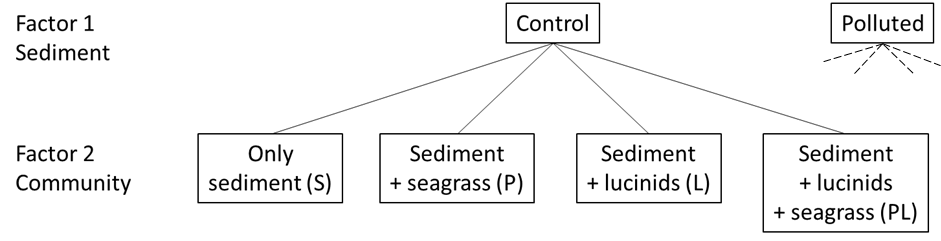


**Figure S1.** Orthogonal experimental design used in the mesocosm experiment with levels of the factors Sediment (Control and Polluted) and Community (S, P, L, PL).


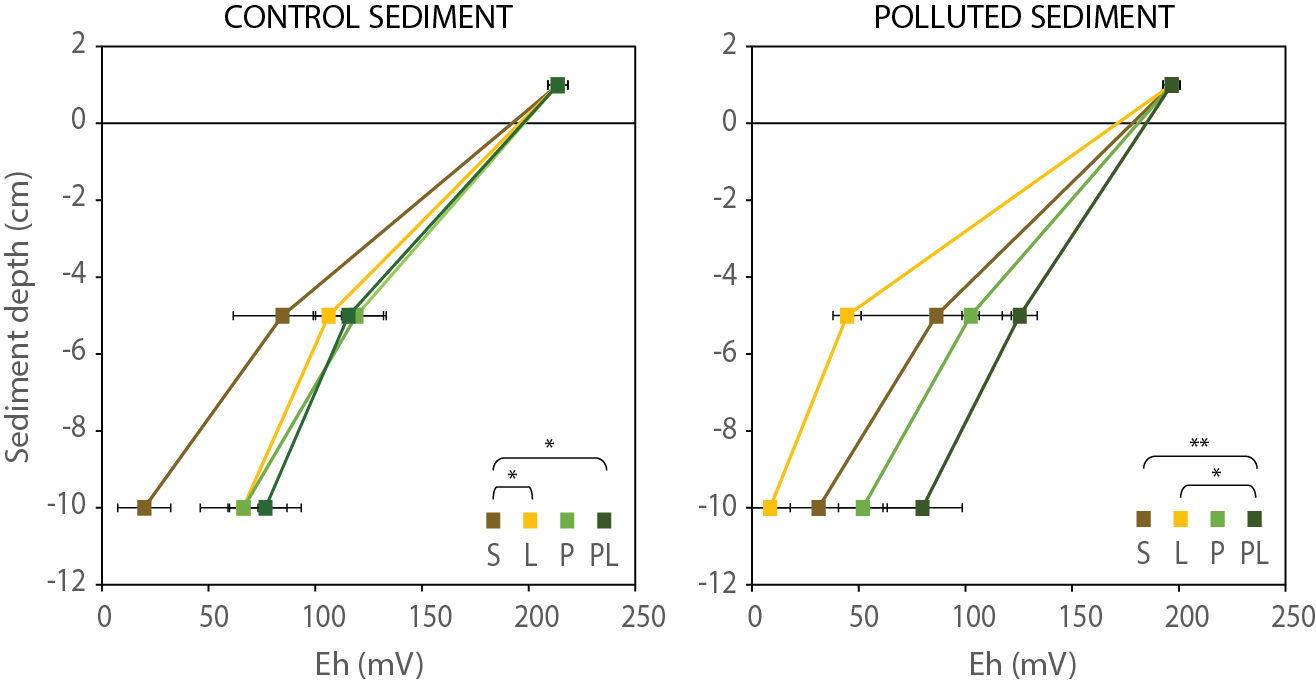


**Figure S2.** Vertical profiles of sediment redox potential (Eh) in sediment porewater at the end of the experiment in control vs polluted sediments. Letters and colours indicate different “Community” levels as reported in the figure legend. Asterisks (*, p<0.05; **, p<0.01) indicate significant differences; see Table S3 for the results of the PERMANOVA pair-wise test.


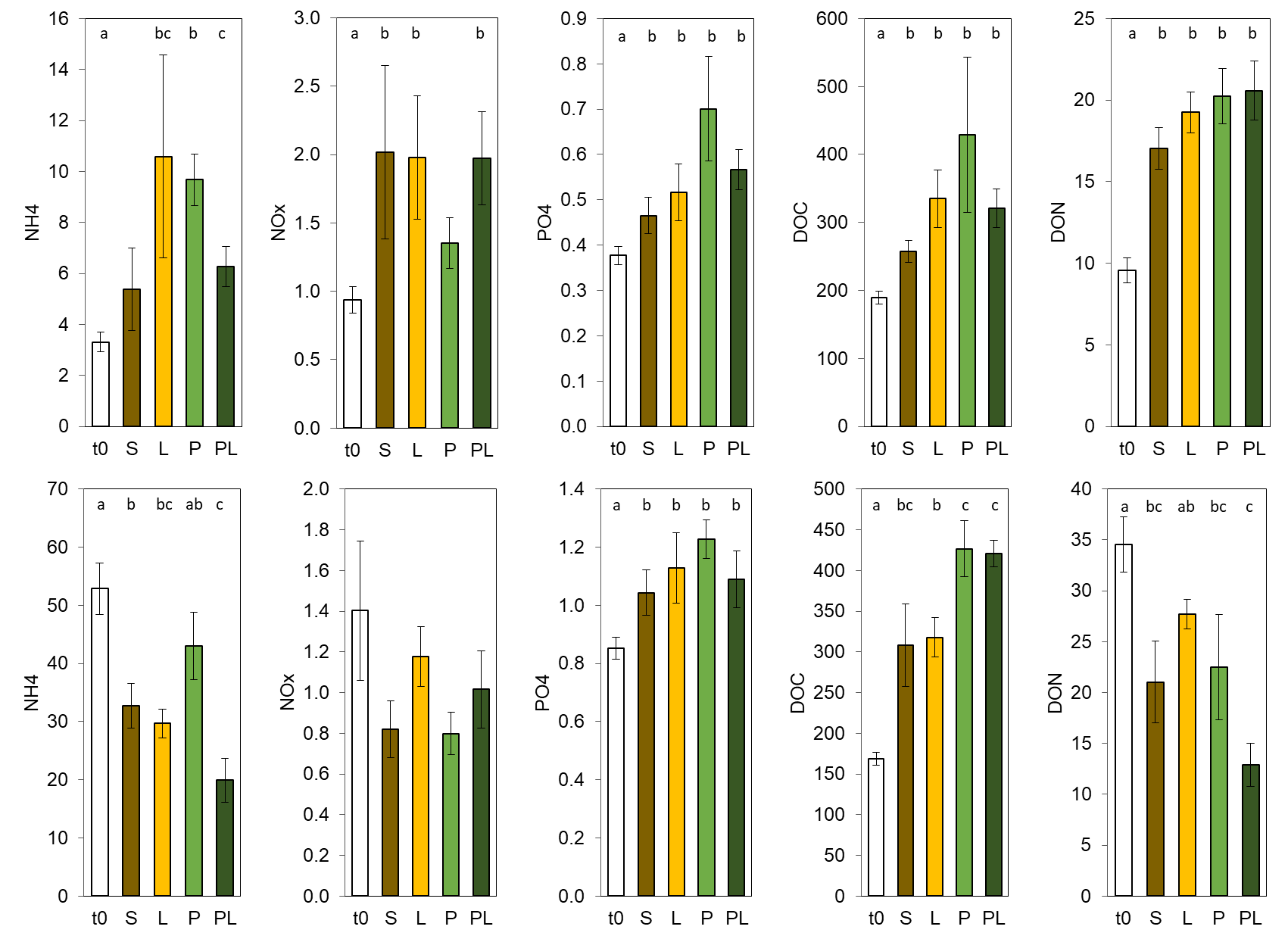


**Figure S3.** Sediment inorganic and organic nutrient concentrations (µM) according to the different treatments. Upper and lower panels are for the control and polluted sediments, respectively. Different letters above the bars represent significant differences as indicated in Table S3; same letters indicate no difference between specific treatments; absence of letters indicates that the specific treatment was no different from all the others.


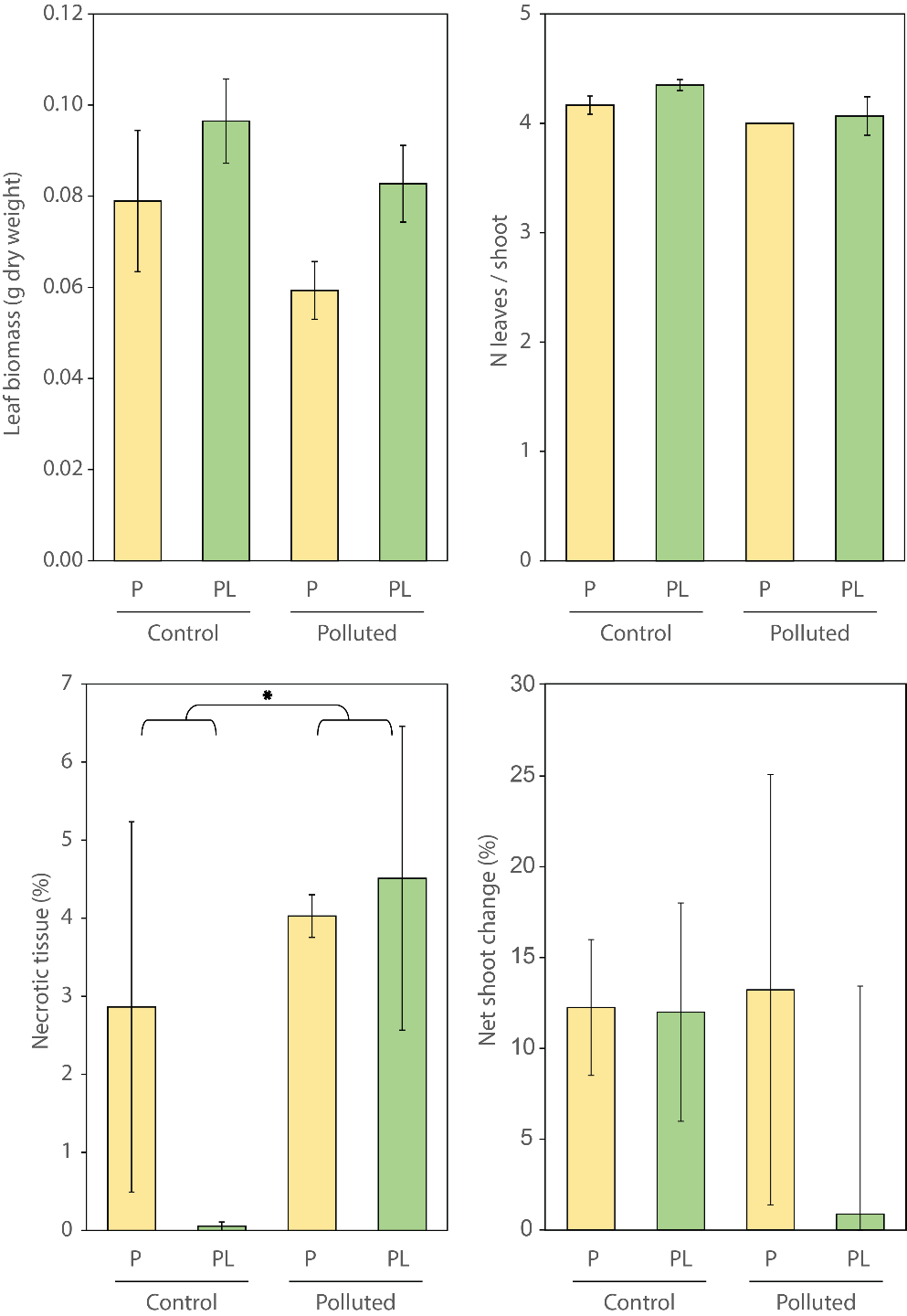


**Figure S4.** Plant growth and morphology. A) Leaves biomass, B) Number of leaves per shoot, C) Percentage of necrotic tissue, and D) Net shoot change of *C. nodosa* at the end of the experiment. Levels of the factor “Community” are identified with letters as indicated in the methods. Colours indicate absence (yellow) or presence (green) of the interaction with lucinid clams. Asterisks (*, p<0.05) indicate significant differences; see Table S5 for the statistics.


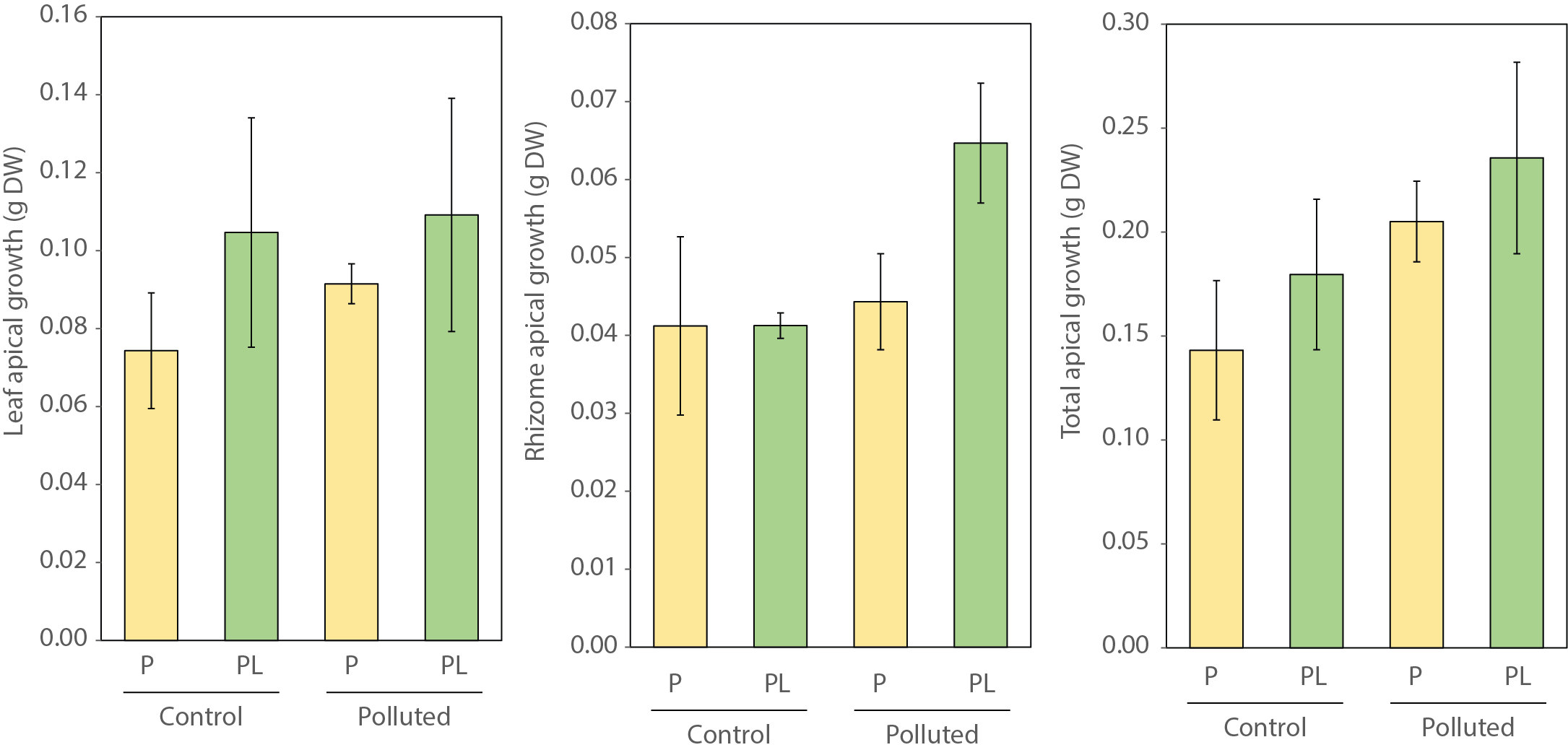


**Figure S5.** Newly produced plant biomass. A) Leaf apical growth, B) Rhizome apical growth, C) Total apical growth of C. nodosa at the end of the experiment. Levels of the factor “Community” are identified with letters as indicated in the methods. Colours indicate absence (yellow) or presence (green) of the interaction with lucinid clams. See Table S6 for the statistics.


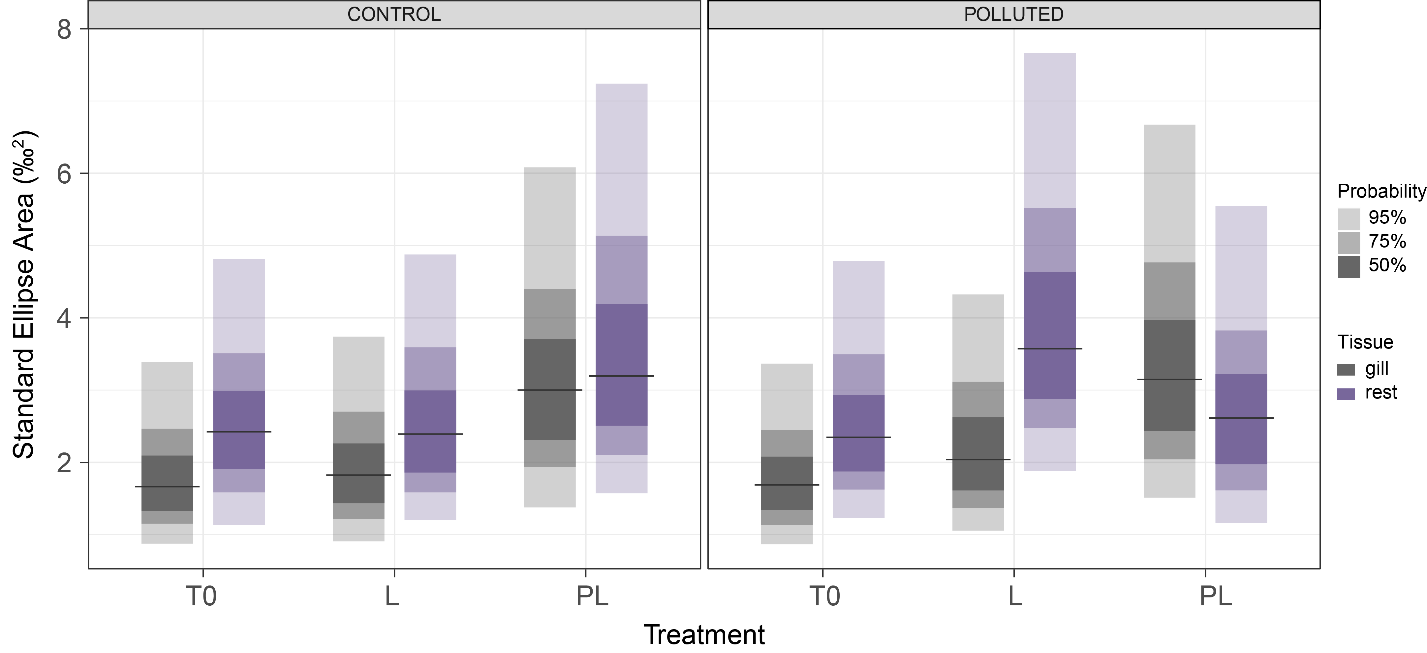


Figure S6. Distribution of Bayesian ellipses (SEA_B_) showing the isotopic niche width (as a proxy of trophic specialization) and its uncertainty, color-coded in purple for symbiont-free (rest) and grey for symbiont-hosting (gill) lucinid clam tissues. Black lines represent the mode while the shaded boxes represent the 50%, 75% and 95% credible intervals from dark to light.
